# Evaluation of *hsp65* Nested PCR-Restriction Analysis (PRA) for Diagnosing Tuberculosis in a High Burden Country

**DOI:** 10.1155/2013/391549

**Published:** 2013-10-24

**Authors:** Sara Macente, Clarice Queico Fujimura Leite, Adolfo Carlos Barreto Santos, Vera Lúcia Dias Siqueira, Luzia Neri Cosmo Machado, Nadir Rodrigues Marcondes, Mario Hiroyuki Hirata, Rosário Dominguez Crespo Hirata, Rosilene Fressatti Cardoso

**Affiliations:** ^1^Post Graduation Program in Biosciences Applied to Pharmacy, Department of Clinical Analyses and Biomedicine, State University of Maringa, 87020-900 Maringá, PR, Brazil; ^2^School of Pharmaceutical Sciences, Department of Biological Sciences, Paulista State University, Araraquara, 14800-901 São Paulo, SP, Brazil; ^3^Department of Clinical Analyses and Biomedicine, State University of Maringa, 87020-900 Maringá, PR, Brazil; ^4^Center of Medical and Pharmaceutical Sciences, State University of Western of Parana, 85819-110 Cascavel, PR, Brazil; ^5^School of Pharmaceutical Science, University of São Paulo, 05508-900 São Paulo, SP, Brazil

## Abstract

Current study evaluated the *hsp65* Nested PCR Restriction Fragment Length Polymorphism Analysis (*hsp65* Nested PCR-PRA) to detect and identify *Mycobacterium tuberculosis* complex directly in clinical samples for a rapid and specific diagnosis of tuberculosis (TB). *hsp65* Nested PCR-PRA was applied directly to 218 clinical samples obtained from 127 patients suspected of TB or another mycobacterial infection from July 2009 to July 2010. The *hsp65* Nested PCR-PRA showed 100% sensitivity and 95.0 and 93.1% specificity in comparison with culture and microscopy (acid fast bacillus smear), respectively. *hsp65* Nested PCR-PRA was shown to be a fast and reliable assay for diagnosing TB, which may contribute towards a fast diagnosis that could help the selection of appropriate chemotherapeutic and early epidemiological management of the cases which are of paramount importance in a high TB burden country.

## 1. Introduction

Tuberculosis (TB) has been known as a major public health challenge worldwide for centuries [1]. The diagnosis of TB is currently based on microscopic detection of acid fast bacilli (AFB) by Ziehl-Neelsen staining and culture of clinical samples. As a positive AFB smear does not always indicate infection by *Mycobacterium tuberculosis*, the differentiation of mycobacteria species is crucial and it depends on the bacillus isolation in culture. Mycobacteria identification in some laboratory, mainly in low income countries, is currently performed by phenotypic and biochemical analyses, which are laborious and time consuming procedures and sometimes do not achieve the precise species identification [2].

The early detection and identification of mycobacteria infection are highly important to provide the adequate treatment in accordance with the species and prevent drug resistance [3, 4]. Moreover, a fast detection and identification of mycobacteria species contribute to efficiently solve epidemiological questions mainly in TB high burden countries [3].

PCR-based methods have been applied for rapid detection of mycobacteria species directly on clinical samples to improve the laboratory diagnosis [5–7]. However, despite being promising, the sensitivity of these methods is sometimes low mainly in samples with negative AFB smear [8]. Also, the development of PCR assays has used different genomic targets, such as IS6110, 16S rDNA, *rpoB*, *recA*, and* hsp65*, which have shown discrepant results in sensitivity [9]. Further, false-positive results in PCR assays for detecting *M. tuberculosis* have been reported [9], suggesting that specificity is also a critical aspect in PCR assays mainly for detection of the IS6110 target [10].

The gene encoding the 65-kDa heat shock protein (*hsp65*) has been reported as a useful target for mycobacteria detection by PCR-based methods. Further fragment analysis of PCR products treated with *BstE*II and *Hae*III endonucleases (Polymerase Chain Reaction Restriction Enzyme Analysis—PRA) allows the differentiation of mycobacteria isolates [11]. PRA is a relatively fast and low cost method without the requirement of specialized equipments, and information on restriction profiles of 113 mycobacterial species are available in a database (http://app.chuv.ch/prasite/index.html) [12].

Modifications of PRA method have been proposed to increase sensitivity and specificity of the assays [2, 13–15], including a nested PCR-PRA protocol reported by Wu et al. [3] for detection of mycobacterial species.

We have evaluated the applicability of the nested PCR-PRA targeting *hsp65* for direct detection of *M. tuberculosis* complex, in clinical samples, in order to contribute to the rapid laboratory diagnosis of TB.

## 2. Material and Methods

### 2.1. Reference Strains and DNA Preparation

The reference strains *M. tuberculosis* H37Rv (ATCC 27294), *M. bovis* AN5, *M. smegmatis *(Central Laboratory-LACEN/PR, Brazil), *M. kansasii* (LACEN/PR, Brazil), *M. fortuitum* (LACEN/PR, Brazil), *M. szulgai* (LACEN/PR, Brazil), *M. massiliense* (LACEN/PR, Brazil), *M. abscessus* (LACEN/PR, Brazil), *M. chelonae* (LACEN/PR, Brazil), and *M. avium* (LACEN/PR, Brazil) were used to evaluate the specificity and sensitivity of Nested PCR-PRA.

Mycobacterial DNA were obtained from a loopful of each reference strain cultured in Lowenstein-Jensen (L-J) medium that was suspended in 300 *μ*L of 6 M guanidine hydrochloride lyses solution (Gibco BRL, Life Technologies, Germany) and submitted to thermal shock by boiling for 10 minutes, followed by ice bath for 15 min. This procedure was repeated three times and samples were left overnight at −20°C. DNA was purified with phenol/chloroform/isoamyl alcohol (25 : 24 : 1, v/v), followed twice by chloroform/isoamyl alcohol (24/1, v/v), and precipitated with absolute ethanol. DNA was solubilized in TE (Tris-HCl 10 mM; EDTA 1 mM, pH 8.0) and stored at −20°C till use.

### 2.2. Clinical Samples and DNA Extraction

The clinical samples were prospectively obtained from 127 patients suspected of mycobacterial infection who were attended at the Municipal Laboratory in Cascavel, Parana, Brazil, from July 2009 to July 2010. The samples were immediately placed in thermal box and sent to the Laboratory for Teaching and Research in Clinical Analyses (LEPAC) at the State University of Maringa (UEM), Parana. The study was approved by the Ethics Committee of the State University of Maringa, Parana (protocol no. 418/2009).

Two hundred eighteen samples (212 sputa, 3 cerebrospinal fluid, 2 bronchial lavage, and 1 plural fluid) were tested using AFB smear (Zielh-Neelsen) and cultured by Ogawa Kudoh method [16]. Twenty-seven patients had positive culture (47 sputa and one bronchial lavage) and the mycobacteria species were identified by phenotypic methods [17].

DNA was extracted by previous treatment of 500 *μ*L of the clinical samples (except for cerebrospinal fluids) with 250 *μ*L of 1% N-Acetyl-L-Cysteine-NaOH (Roche Diagnostics, Mannheim, Germany), vortex stirred, and left at room temperature for 15 min. Samples were centrifuged at 12,000 g for 5 min and DNA was extracted as described for reference strains. Cerebrospinal fluid samples were boiled for 10 min, centrifuged at 12,000 g for 5 min, and the supernatant was separated to be further analyzed. All DNA samples were stored at −20°C till use.

### 2.3. Nested PCR

The *hsp65* Nested PCR assay was performed according to Wu et al. [3]. First amplification was carried out with specific primers for *Mycobacterium* spp M1 (5′-CCCCACGATCACCAACGATG-3′) and M4 (5′-CGAGATGTAGCCCTTGTCGAACC-3′) (Invitrogen-Integrated DNA Technologies, Inc., Coralville, USA) which generated a 463-bp product. PCR assays had 1 *μ*L of template (5 *μ*L for cerebrospinal fluid) in 24 *μ*L (20 *μ*L for cerebrospinal fluid) of reaction mixture containing 0.2 *μ*M of each primers (Integrated DNA Technologies, Inc., Coralville, USA) and PCR Master Mix (Promega Corporation, Madison, Wisconsin, USA) according to manufacturer's instructions. Nested PCRs were performed using 5 *μ*L of the first amplification in 45 *μ*L of reaction mixture containing 0.2 *μ*M of each primer TB11 (5′-ACCAACGATGGTGTGTCCAT-3′) and TB12 (5′-CTTGTCGAACCGCATACCCT-3′) [9] (Invitrogen-Integrated DNA Technologies, Inc., Coralville, USA) and PCR Master Mix (Promega Corporation, Madison, Wisconsin, USA) according to manufacturer's instruction, which generated a 440-bp product. All amplifications were carried out in a PERKIN-ELMER Gene Amp PCR System 2400 (Waltham, Massachusetts, USA). Positive, negative, and inhibitor controls were included in all PCR assays. Adequate care was taken to prevent contamination. All PCR steps were carried out in separate rooms (clean reagents, extraction, and amplification rooms).

### 2.4. Nested PCR Sensitivity

Assessment of Nested PCR sensitivity and intra-assay reproducibility (M1, M4, and TB11 and TB12 primers) was firstly performed using DNA extracted from the reference strains (tenfold serial dilution 100 *μ*g to 100 fg) in triplicate. Also, the sensitivity and interassay reproducibility were determined directly in mycobacterial spiked sputum. Freshly mycobacterial suspensions of each reference strains were prepared according to 1 McFarland turbidity and serial dilutions (10^−1^ to 10^−20^) were carried out in triplicate. Afterwards, 50 *μ*L of each bacterial dilution were spiked in 450 *μ*L of the homogenized sputum, previously determined as negative AFB smear and culture negative for mycobacteria [9]. DNA extraction previously described for clinical samples was carried out for each triplicate mycobacterial spiked sputum (dilutions 10^−1^ to 10^−20^). PCR inhibitors controls for each dilution in spiked sputum were performed in the standardization step by the addition of 1 *μ*L (6.25 ng) of *M. tuberculosis* H37Rv DNA to 25 *μ*L of each extracted dilution spiked sputum in specific and separate rooms to avoid possible false positive. All procedures were carried out with essential care and using barriers tips.

### 2.5. PRA Analysis

The Nested PCR products were digested with *BstE*II (Sigma-Aldrich Corporation, St. Louis, Missouri, USA) and *Hae*III (Promega Corporation, Madison, Wisconsin, USA) endonucleases, following manufacturer's instructions. Restriction products were analyzed by agarose gel electrophoresis (4% METAGEL, Pronadisa, Madrid, Spain) and stained with ethidium bromide solution 1.0 g/mL. The band sizes were estimated with 25 and 50 bp DNA ladders (Invitrogen-Integrated DNA Technologies, Inc., Coralville, USA) as the molecular size standards. The PRA patterns were compared to ones reported in PRASITE (http://app.chuv.ch/prasite/index.html).

### 2.6. Data Analysis

Amplification sensitivity and specificity were first determined using DNA of reference strains and with mycobacterial spiked sputum. After, these parameters were determined in clinical samples analyses separately. Samples were considered positive by *hsp65* Nested PCR when a single band of DNA (440-bp) was observed; then the species were identified by PRA. Negative results were considered in the absence of specific amplification after no detection of inhibitor. The sensitivity and specificity of the assay using clinical samples were compared with AFB smear and culture (gold standard) and expressed in percentage (95% confidence interval). Proportion of positive and negative results were compared using the Fisher's Exact Test. Test with *P* values <0.05 was considered statistically significant. Statistical analysis was done by OpenEpi software version 2.3.1 (http://www.openepi.com/v37/Menu/OE_Menu.htm).

## 3. Results and Discussion

In this study, we evaluated the feasibility of applying *hsp65* Nested PCR-PRA to detection and identification of mycobacteria in 218 clinical samples from 127 patients undergoing TB suspected infection by comparison with AFB smear and culture.

Early diagnosis of TB and differentiation of *M. tuberculosis* complex from Nontuberculous Mycobacteria (NTM) in clinical samples are of paramount importance for proper clinical and epidemiological management, since most NTM are resistant to drugs commonly used in TB treatment [4] and patients who are suspected to have TB have to be placed in isolate room immediately [3]. Also, the early identification of NTM is very important considering many of them are now recognized as true pathogens in important human infections [18] and their incidence has been increasing [19–21].

To circumvent the difficulties in identification of mycobacteria species by conventional methods, the PCR-PRA developed by Telenti et al. [11] became a good alternative. Wu et al. [3] applied for the first time, which we have knowledge, the PCR-PRA directly in clinical samples for differentiation of *M. tuberculosis* complex from NTM and improved the detection limit by adding a Nested PCR to the assay.

In the present study, both *hsp65* Nested PCR with DNA from reference strains as in sputum sample spiked with serial dilutions of reference strains showed reproducibility. The assay allowed detection limit of 1 ng of mycobacterial DNA by using serial dilution of all reference strains DNA. The sensitivity of the Nested PCR applied to spiked sputum was 10^−3^ dilution, equivalent to approximately 5,000 mycobacterial cells for all reference strains.

The *hsp65* Nested PCR detection limit of mycobacterial cells in spiked sputum samples observed in current study (5,000 mycobacterial cells) was lower than obtained by Wu et al. [3], but this did not affect the sensitivity of the test applied to clinical samples when compared to AFB smear (*P* < 0.05) and culture (*P* < 0.05).

The sensitivity of *hsp65* Nested PCR-PRA carried out in clinical samples, used in present study, compared with microscopy and culture was 100% (26/26 and 27/27, resp.). Specificity and positive predictive value were 93.1% (94/101) and 78.8% compared with microscopy and 95.0% (95/100) and 84.4% compared with culture (*P* < 0.05), respectively (Table 1).

All 27 mycobacteria isolated from 26 AFB smear positive and one AFB negative bronchial lavage samples were identified as *M. tuberculosis* by phenotypic methods [17] and *hsp65* PCR-PRA [3].

*hsp65* Nested PCR-PRA applied to all culture positive clinical samples was positive for *M. tuberculosis* complex (*Hae*III—150, 130, 70 bp—and *BstE*II—235, 120, and 85 bp). False-negative or inconclusive results by *hsp65* Nested PCR-PRA were firstly observed in 11 sputa samples (9 AFB smear 1+ and 2 AFB smear 3+) when the single-PCR was applied to direct detection of mycobacteria in sputa samples but was positive by *hsp65* Nested PCR showing a 440-bp product. False-negative or inconclusive results among samples that showed AFB smear 1+ and occasionally 3+ found in the first PCR were also reported by Wu et al. [3]. In a study with a single-PCR, Kim et al. [14] also found such low sensitivity in the detection of mycobacteria in AFB smear 1+ sputum sample. Quantity of DNA target in clinical samples was probably insufficient to produce a visible DNA band in single-PCR agar gel analysis. Consequently, Nested PCR was an important strategy for increasing the sensitivity of the method used in present study as observed previously by Wu et al. [3].

Of 101 patients (171 clinical samples) showing negative mycobacterial culture and AFB smear, there were five (9 sputa samples and one bronchial-alveolar washing sample) that showed inconsistent results with *hsp65* Nested PCR-PRA. In three of these patients with pulmonary symptoms (two or three sputa samples of each one) positive result for *M. tuberculosis* by *hsp65* Nested PCR-PRA were observed. The possibility of active TB had been firstly ruled out considering the negative culture results. The positive *hsp65* Nested PCR-PRA results, for these patients, cannot be ruled out once very low amount of mycobacteria DNA, originating from an early TB infection, may have occurred and only the *hsp65* Nested PCR-PRA detected the bacillus. As these patients were homeless and did not return to the health care facility for monitoring the case, the diagnosis was not concluded and this may be a bias in the study. The fourth patient was in use of chemotherapy for breast cancer and had bilateral pulmonary infiltrates. For the fifth patient (two sputa samples obtained in different days with negative AFB smear and culture) the single-PCR and Nested-PCR for both samples were positive (463-bp and 440-bp products, resp.). In these two samples, PRA analysis identified *M. immunogenum* type 2 (*Hae*III—200, 70, 60, 55 bp—and *BstE*II—320, 130 bp). According to Wilson et al. [22] this species fails to grow in L-J medium and it may have occurred by culturing on Ogawa medium used in the current study. Besides, negative AFB smear observed is consistent with the low sensitivity of the method (approximately 20 to 50% of patients with pulmonary infection are negative AFB smears) [23]. This species occurs in the environment and when associated with infectious processes, it is closely related to hospital contaminations with contaminated water [22]. In our study we could not establish the association of the positive result by Nested PCR-PRA with the disease.

It is noteworthy that all PCR may lead to a false negative result that can be caused by DNA polymerase inhibitors present in the clinical sample. To overcome this issue, in our study, was included in the spiked sputum an inhibitor control by adding *M. tuberculosis* DNA in the extracted sample. To avoid possible contamination, leading to false positive results, that step was carried out by handling all samples firstly and after the addition of *M. tuberculosis* DNA was done in a separate room. For patients' clinical samples study, the inhibitor control was carried out only for sample, which resulted in negative Nested PCR. However, the use of an internal amplification control using a nontarget DNA sequence present in the genome of the microorganism to be detected is of common agreement and has to be performed separately to overcome this matter.

PCR-based protocols have been suggested for differentiating *Mycobacterium* sp in culture or directly in clinical sample. Kim et al. [24] proposed a multiprobe Real-Time PCR targeting the *hsp65* gene, the same target used in our study, which showed a sensitivity level of 94.3%. *hsp65* Nested PCR-PRA, used in or study, showed 100% sensitivity. Certainly the higher sensitivity was consequent by the second step of amplification, which according to the above authors this step would improve the sensitivity in their study too. One important advantage of the Real Time method is it can detect coinfection that can be underestimated by culture and misunderstood by *hsp65* Nested PCR-PRA.

## 4. Conclusions

The combination of *hsp65* Nested PCR and PRA seems to be a powerful technique for fast detection and identification of mycobacteria at species level mainly in case of TB where an early diagnosis is very important to start as soon as possible adequate therapy and establish prevention measures for TB control. In our experience, the *hsp65* Nested PCR-PRA shows advantages in time to release the result that is 24–48 hours for detection and identification of *M. tuberculosis* complex, whereas phenotypic tests require at least four to eight weeks for final mycobacteria identification. Even when automated culture is used, the diagnosis is too time consuming. Regarding the application of *hsp65* Nested PCR-PRA in laboratory routine for TB diagnosis, it may be stated that the method is cost-effective, fast, and does not require any expensive equipment or very technical expertise when compared to biochemical tests, real-time PCR, sequencing, or high performance liquid chromatography (HPLC). Data shows that *hsp65* Nested PCR-PRA may be an important tool for diagnosing TB in low income countries with high incidence of the disease, where a faster result for immediate therapy is of importance for epidemiological control.

## Figures and Tables

**Table 1:** Detection of *Mycobacterium* spp by *hsp65 *Nested PCR-PRA, culture, and AFB smear.

	*hsp65* Nested PRC-PRA			
	Positive (%)	Negative (%)	Total (%)	*P* value
Culture				<0.0000001
Positive	27 (21.25; 95% CI 15.04–29.16)	0 (0.0; 95% CI 0.0–2.94)	27 (21.25; 95% CI 15.04–29.16)
Negative	5 (3.94; 95% CI 1.69–8.89)	95 (74.80; 95% CI 66.6–81.55)	100 (78.74; 95% CI 70.84–84.96)
Total	**32 (25.20; 95% CI 18.45–33.4)**	**95 (74.80; 95% CI 66.6–81.55)**	**127 (100.0; 95% CI 97.06–100)**
AFB* smear				<0.0000001
Positive	26 (20.47; 95% CI 14.37–28.31)	0 (0.0; 95% CI 0.0–2.94)	26 (20.47; 95% CI 14.37–28.31)
1+	11 (8.66; 95% CI 4.91–14.84)		
2+	7 (5.51; 95% CI 2.70–10.94)		
3+	8 (6.30; 95% CI 3.23–11.94)		
Negative	7 (5.51; 95% CI 2.70–10.94)	94 (74.02; 95% CI 65.76–80.86)	101 (79.52; 95% CI 71.69–85.63)
Total	**33 (25.98; 95% CI 19.14–34.24)**	**94 (74.02; 95% CI 65.76–80.86)**	**127 (100.0; 95% CI 97.06–100)**

*AFB: acid fast bacilli.
